# Case report: Serious unexpected vascular events in two patients with lymphocytic variant hypereosinophilic syndrome

**DOI:** 10.3389/fcvm.2023.1256862

**Published:** 2023-09-27

**Authors:** Nathan Torcida, Giulia Casalino, Antoine Bondue, Lise Jodaitis, Frederic Vanden Eynden, Florence Roufosse

**Affiliations:** ^1^ Department of Neurology, Hôpital Universitaire de Bruxelles-Hôpital Erasme, Brussels, Belgium; ^2^ Department of Cardiology, Hôpital Universitaire de Bruxelles-Hôpital Erasme, Brussels, Belgium; ^3^ Department of Cardiac Surgery, Hôpital Universitaire de Bruxelles-Hôpital Erasme, Brussels, Belgium; ^4^ Department of Internal Medicine, Hôpital Universitaire de Bruxelles, Hôpital Erasme, Université Libre de Bruxelles, Brussels, Belgium

**Keywords:** stroke, arterial dissection, hypereosinophilc syndrome, coronary aneurism, lymphocytic variant hypereosinophilic syndrome, valsalva sinus aneurysm

## Abstract

**Background:**

Lymphocytic-variant hypereosinophilic syndrome (L-HES) is a form of reactive hypereosinophilia, most commonly associated with interleukin-5 over-production by clonal, most commonly CD3^−^CD4^+^CD2^hi^CD5^hi^CD45RO^+^ T-cells. Patients often present with predominant cutaneous and soft-tissue manifestations, while cardiovascular involvement is uncommon.

**Methods:**

We reviewed the medical files of two L-HES patients followed in our center who developed serious vascular complications and performed a literature review for similar cases.

**Results:**

Patient 1, a 52-year-old female, presented with an ischemic stroke secondary to left middle cerebral artery dissection after 10 years of indolent L-HES. Blood eosinophilia was controlled with oral corticosteroids (OCS), but OCS-tapering attempts with hydroxyurea and pegylated interferon failed, prompting the introduction of mepolizumab with rapid normalization. Patient 2, a 62-year-old female, had been asymptomatic for 10 years without treatment when a NSTEMI occurred, due to coronary artery occlusion secondary to a large cauliflower-aneurysm of the proximal aorta and aneurysmal dilatation of several coronary arteries, requiring semi-urgent surgical management. Aortic wall staining for eosinophil major basic protein showed eosinophils in the adventitia. Blood eosinophilia was controlled with OCS.

**Conclusions:**

Patients with apparently clinically benign L-HES may develop arterial complications, consisting in dissection and/or aneurysm dilatation of medium-to-large vessels with serious consequences. The value of performing regular vascular imaging and monitoring during follow-up has yet to be determined.

## 1. Introduction

Hypereosinophilic syndrome (HES) is characterized by persistent and marked blood and tissue eosinophilia associated with eosinophil-mediated organ damage and/or dysfunction (1). Cardiovascular complications account for significant HES-related morbidity and mortality (2). In addition to direct eosinophil-mediated damage to the heart, coronary vasospasm, arterial and/or venous thrombi as well as microvascular damage may occur (3). In lymphocytic variant (L-) HES, hypereosinophilia is secondary to interleukin-5 over-production by clonal, most commonly CD3^−^CD4^+^CD2^hi^CD5^hi^CD45RO^+^ T-cells. This benign and indolent lymphoproliferative disease is driven by mature type 2 CD4 T-cells that are typically found in the blood, lymph nodes, and skin (4), accounting for the predominant cutaneous and soft-tissue manifestations observed in these patients. Prognosis is generally considered favorable, with infrequent cardiovascular involvement, although a small proportion of patients progress to T-cell lymphoma (4). Herein we report two patients with long-standing stable L-HES, who developed serious complications due to vascular injury.

## 2. Methods

Medical records of patients were reviewed according to local ethics committee guidelines and a systematic literature review was conducted using the PubMed database. To formulate the search strategy, relevant keywords and Medical Subject Headings (MeSH) terms were identified to cover vascular complications among HES patients. The search equation was developed by combining these terms using Boolean operators (Supplementary Material). An initial search was performed to evaluate the relevance and specificity of the search equation. Based on the outcomes of the initial search, the search equation was refined. A full search was conducted using the refined search equation, and the search results were screened for L-HES vascular complications. Articles were selected for inclusion based on a full-text assessment of their relevance. The search strategy yielded a total of 77 articles, of which 3 articles met our criteria and were therefore included.

## 3. Results

**Figure 1:**
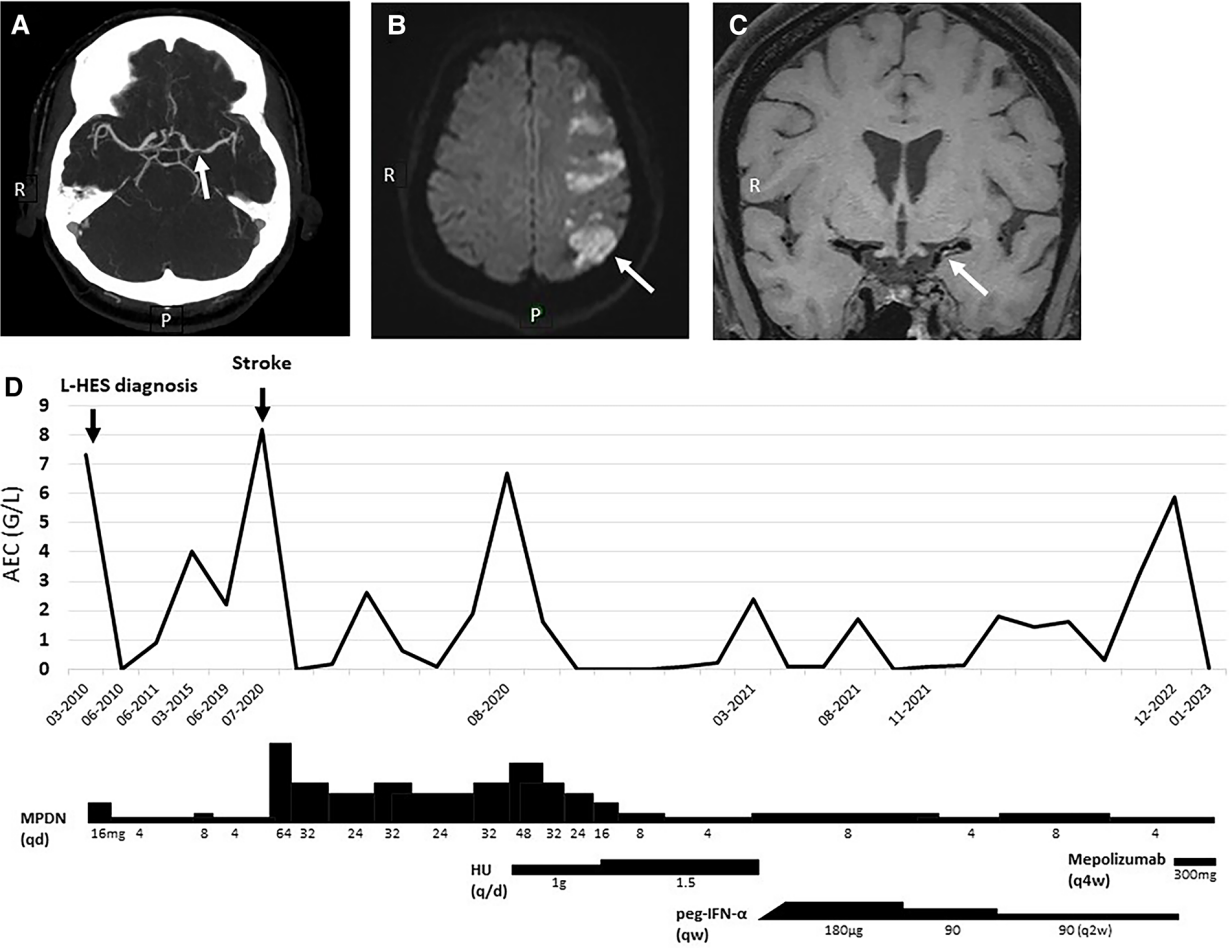
Patient 1 cerebrovascular imaging studies, blood eosinophil counts, and HES treatment. (**A**) Cerebral CT-scan showing left middle cerebral artery irregularities with “flute-beak” appearance. (**B,C**) Diffusion-weighted cerebral magnetic resonance showing cortico-subcortical left hemispheric infarction, and intramural hypersignal on T1 sequences of the left middle cerebral artery suggesting presence of a vessel-wall hematoma. (**D**) Temporal evolution of AEC and therapy. AEC, absolute eosinophil count; HU, hydroxyurea; MPDN, methylprednisolone; peg-IFN-α, pegylated interferon-alpha; qd, every day; qw, every week; q2w/4w, every two/four weeks.

Patient 1: A 52-year-old woman diagnosed with L-HES in 2010 developed aphasia and right brachiofacial paresis in July 2020. Cerebral computed tomography (CT)-scan showed a cortico-subcortical left hemispheric infarction, and magnetic resonance (MR)-angiography with vessel wall imaging sequences suggested dissection of the left middle cerebral artery (Figures 1A–C). Her blood absolute eosinophil count (AEC) upon admission was 8.18 G/L.

Her past HES history started with the fortuitous discovery of asymptomatic blood hypereosinophilia in 1995, followed by the development of pruritus, facial oedema, and Raynaud’s with toe chilblain in 2010, at which time the diagnosis of L-HES was made in presence of an AEC at 7.35 G/L and a circulating CD3^−^CD4^+^ T-cell population (1.9% of lymphocytes, absolute count 0.034 G/L). Oral corticosteroid (OCS) therapy was initiated, with symptom improvement and normalization of AEC. Treatment was progressively tapered, with persistent clinical remission at 4 mg methylprednisolone (MPDN) daily although hypereosinophilia recurred. Her CD3^−^CD4^+^ T*-*cell counts were stable over time, and cardiovascular assessments including angiography of lower extremities and repeated echocardiography and cardiac MR were normal.

Upon admission for stroke, HES was considered the most likely cause of vascular dissection after an extensive workup. Treatment with aspirin (80 mg/day) and atorvastatin (40 mg/day) was started in accordance with local protocols for the management of acute ischemic stroke. The daily OCS dose was increased and AEC normalized within 24 h (Figure 1D). Her neurological symptoms resolved completely within months. Hypereosinophilia recurred with OCS-tapering, so classical second-line agents were introduced for OCS-sparing purposes. Neither hydroxyurea nor sub-cutaneous pegylated interferon-alpha (peg-IFN-α) achieved this goal. She recently started mepolizumab 300 mg/4 weeks, leading to a dramatic reduction of her AEC to 0.01 G/L.

**Figure 2:**
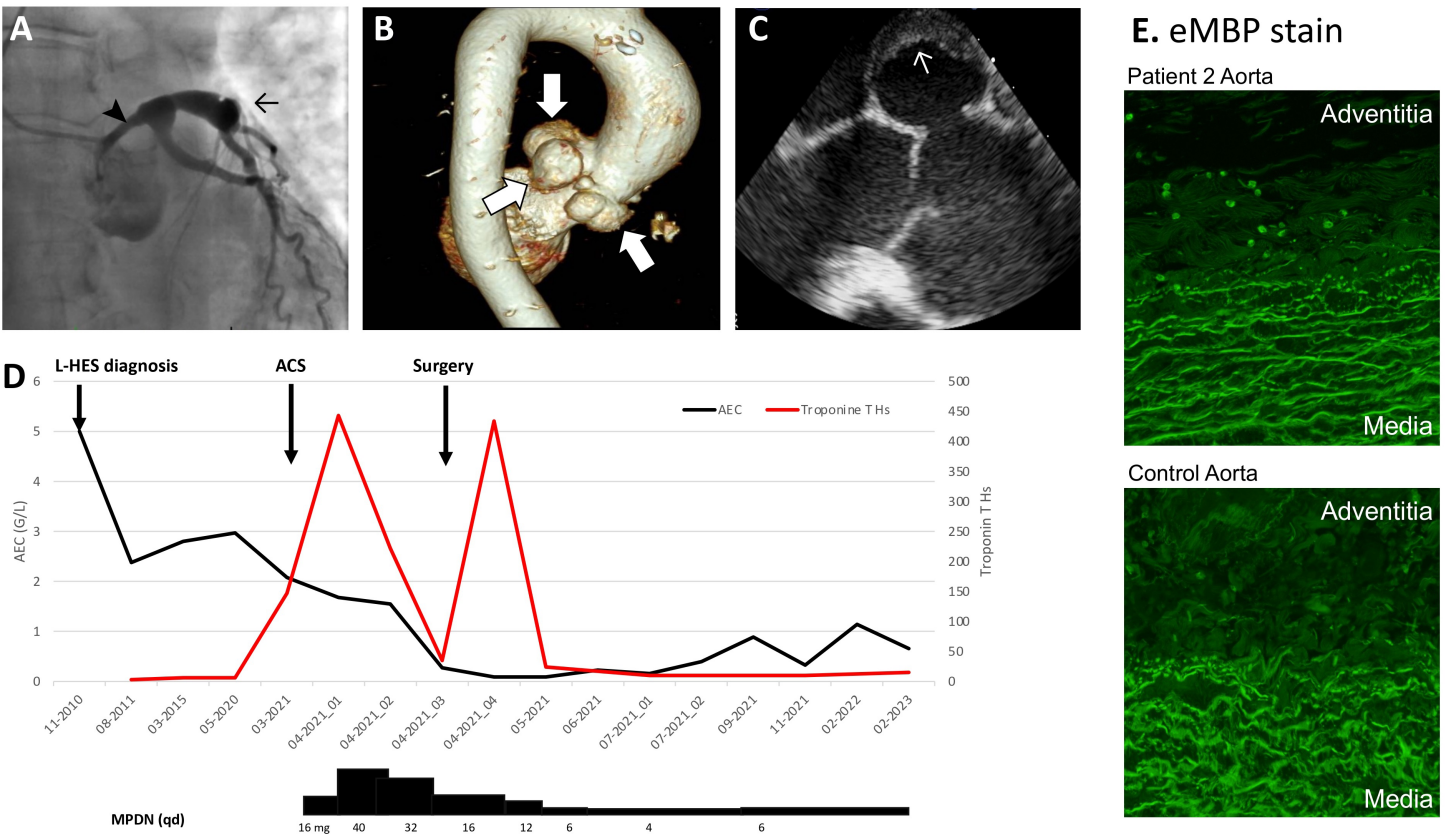
Patient 2 cardiovascular imaging and histology, blood eosinophil counts, and HES treatment. (**A**) Coronary angiography showing aneurysmal dilation of CA (→) and extrinsic compression of the left main trunk by a VS aneurysm (➤). (**B**) Aortic MR-angiography showing giant polylobulated aneurysms occupying the non-coronary and the left VS. (**C**) Perioperative transesophageal echocardiography showing mural thrombus in a VS aneurysm. (**D**) Temporal evolution of AEC, Troponin T_hs_ and therapy. (**E**) Immuno-fluorescent staining for eMBP-1 on resected aorta from patient 2 and from a control normo-eosinophilic subject with an aortic dissection, showing intact eosinophils (8 per high power field) and patchy extracellular granule protein deposition in patient 2’s aortic adventitia. Prominent non-specific staining was observed in the media in both patient and control, with a similar pattern to control staining with rabbit immunoglobulin (not shown). 100X magnification. ACS, acute coronary syndrome; AEC, absolute eosinophil count; CA, coronary arteries; CT, computed tomography; eMBP-1, Eosinophil major basic protein; OCS, oral corticosteroid; VS, Valsalva sinus.

Patient 2: A 62-year-old woman diagnosed with L-HES in 2010 experienced constrictive chest pain at rest in March 2021. Myocardial damage was suspected based on increased serum Troponin T (443 ng/L, normal value <14 ng/L) followed by an initial progressive decrease. Her blood AEC was 2.08 G/L. An echocardiography showed severe left ventricular (LV) systolic dysfunction with an ejection fraction of 30% and apical ballooning. Coronary angiography showed proximal aneurysmal dilatation of all three coronary arteries (CA), with compression of the left main trunk by a Valsalva sinus (VS) aneurysm (Figures 2A–C). CT-angiography of the aorta revealed giant polylobulated aneurysms occupying the non-coronary VSs; the largest aneurysm, in the left VS, measured 3.8 cm.

Her past L-HES history was notable for pruriginous bullous skin lesions in 2010, associated with blood hypereosinophilia (4.47 G/L) and a CD3^−^CD4^+^ T-cell population (7% of total lymphocytes, 0.138 G/L). Because the skin lesions regressed spontaneously, she was not treated, and both her AEC (2–3.7 G/L) and her aberrant T-cell subset remained stable over time. Cardiac assessments including yearly electrocardiogram, echocardiography, and serum Troponin were normal until January 2021, when ectasia of the aortic root and VS enlargement were noted.

Hypereosinophilia was considered the likely cause of vascular injury and MPDN was initiated (Figure 2D). In parallel, the initial management of non-ST-elevation myocardial infarction included treatment with atorvastatin (40 mg/day), aspirin (80 mg/day), clopidogrel (75 mg/day), lisinopril (2.5 mg/day) and propranolol (60 mg/day). Hypereosinophilia resolved after 3 days and cardiac function stabilized, allowing a mechanical Bentall procedure with triple aorto-coronary bypass to be performed on day 10. Dehiscence between the aortic annulus and the aorta was noted, explaining pseudo-aneurysmal formation. Intraoperative transesophageal echography showed mural thrombi in the VS, representing a likely source of embolism, consistent with the ischemic injury downstream to the left anterior descending artery. Eosinophil major basic protein (eMBP1) (Figure 2E) and eosinophil derived neurotoxin (EDN) (not shown) staining by indirect immunofluorescence showed intact eosinophils (6–8/high-power-field) and patchy extracellular deposits in the adventitia of the resected proximal aorta. She is currently OCS-dependent with poor tolerance, and we plan to introduce mepolizumab. Clinically, she has recovered normal cardiac function, and serum Troponin T is normal.

Our literature review identified three other cases of severe vascular complications occurring in L-HES patients, either as unique case reports (5–6) or among complications mentioned in a L-HES patient cohort (7) (Supplementary Table S1).

## 4. Discussion

Development of serious vascular complications such as arterial dissection and/or aneurysmal dilatations is uncommon in patients with HES, and in L-HES only 3 cases have been reported besides ours (5–7) (Supplementary Table S1). Our observation suggests that patients with L-HES and uncontrolled eosinophilia may develop serious ischemic complications due to direct damage of the vascular wall of medium-to-large vessels many years after diagnosis, even in the setting of otherwise benign disease presentations.

Neither of our patients were at significant risk for vascular disease (Supplementary Table S1), and the observed presence of scattered eosinophils and eosinophil granule protein deposition in the adventitia of the resected aorta of patient 2 strongly suggests that eosinophils contributed to endovascular damage. Eosinophilic infiltrates have previously been reported in a patient with idiopathic HES who developed CA and VS aneurysms (8) and in the CA adventitia in 8 patients with spontaneous CA dissection (9), but our report is the first to show eosinophil granule protein staining in this setting. Proposed mechanisms for eosinophil-associated vasculopathy include local release of cytotoxic granule proteins (major basic protein, eosinophil cationic protein, eosinophil peroxidase, eosinophil derived neurotoxin) by infiltrating eosinophils and/or endovascular deposition of free circulating granule proteins (8). In addition, activated eosinophils release an array of other mediators that may contribute to functional perturbations and structural damage in their environment, including reactive oxygen species, eicosanoids such as LTC4, pro-inflammatory and pro-fibrotic cytokines, and galectin-10 that forms Charcot Leyden crystals (10). In the setting of L-HES, vascular adhesion and trans-endothelial migration of eosinophils may be favored by the cytokine profile of pathogenic CD3^−^CD4^+^ T-cells. Indeed, these cells produce IL-4, IL-13, and TNF-alpha (11), all known to increase endothelial cell expression of VCAM-1 (that interacts with eosinophil-expressed VLA-4) and CCL26/eotaxin-3 (a potent chemokine for CCR3-expressing eosinophils). Interestingly, digital gangrene has been reported in several patients with CD3^−^CD4^+^ T-cells before L-HES was caracterized (12). Although the causal role of eosinophils in vascular damage remains to be firmly established in the setting of HES, once this complication occurs, AECs should be well controlled by treatment thereafter to prevent recurrence.

Our observation further underlines how unpredictable HES disease course can be and raises the question of how such rare but serious vascular complications can be detected before irreversible damage occurs. Currently, only repeated cardiac assessments (electrocardiogram, echocardiography and serum Troponin T) are recommended to detect cardiovascular complications in HES (2). Our observation underscores that attention should be paid both to the heart and adjoining vascular structures during yearly echocardiography. In HES patients with persistent hypereosinophilia, yearly cardiac MR may increase the likelihood of detection. In addition, vascular imaging studies should be performed promptly in patients with clinical manifestations suggesting possible vasculopathy, and repeated in those with a history of vascular injury to detect involvement of other vascular beds. Inclusion of systematic vascular imaging among follow-up assessments in patients whose eosinophil counts are persistently elevated may be worth considering. CT-angiography has a higher resolution than MR-angiography but because of significant irradiation, MR-angiography is more appropriate for screening.

In conclusion, dissection and (pseudo)aneurysmal formation of medium to large-caliber vessels are uncommon but serious complications of HES. Screening for these complications could be of clinical interest, but the modalities remain to be defined.

## Data Availability

The original contributions presented in the study are included in the article/**Supplementary Material**, further inquiries can be directed to the corresponding author.
