# Supplementary material for: Case report: Serious unexpected vascular events in two patients with lymphocytic variant hypereosinophilic syndrome
Source: Front Cardiovasc Med. 2023 Sep 27;10:1256862. doi: 10.3389/fcvm.2023.1256862 (PMC10565030; doi:10.3389/fcvm.2023.1256862)
Supplement: Supplementary file 1 [file Datasheet1.pdf]

Supplementary Table S1. Severe vascular complications in patients with lymphoid variant hypereosinophilic syndrome.

	Patient 1	Patient 2	Divanji et al [5]	Van Gaalen et al [6]	Lefevre et al (Patient 11) [7]
**Patient History prior to vascular event**					
Sex	F	F	F	F	F
HES History	Blood HE discovered fortuitously during pregnancy at age 27 (5.07 G/L).<br>Pruritus, periorbital edema and Raynaud's at 42. AEC 7.35 G/L.<br>Diagnosis of L-HES ($CD3^{-}CD4^{+}$) at age 42.	Blood HE noted at age 48 (1.79 G/L).<br>Bullous skin lesions containing eosinophilic infiltrates at 49. AEC 4.47 G/L. Repeated episodes, lasting several weeks with spontaneous definitive resolution after 2 years.<br>Diagnosis of L-HES ($CD3^{-}CD4^{+}$) at age 51.<br>Raynaud's at age 55.	Blood HE noted a decade before acute event (~28 yrs-old).<br>Eczema.<br>Temporal artery thrombotic aneurysm; resected (pathology: perivascular eosinophilic infiltrate). Diagnosis: epitheloid hemangioendothelioma.<br>Asymptomatic until acute event, when L-HES was diagnosed ($CD3^{-}CD4^{+}$).	Blood HE noted at age 7.<br>Pruriginous skin lesions, diffuse angioedema, arthralgia, Raynaud's, eosinophilic fasciitis, hepatomegaly, splenomegaly at age 15. AEC 3.87 G/L.<br>Diagnosis of L-HES ($CD3^{dim}TCRVb4^{dim}CD4^{+}$) at age 15.	Blood HE first noted at age 20. Peak AEC 3 G/L.<br>Asthma, eczema, pruritus, superficial lymphadenopathy.<br>Diagnosis of L-HES ($CD3^{-}CD4^{+}$) at age 38.
Maintenance HES therapy	MPDN 4mg/d	None	None	PDN 5 mg/d, IFNα 4x10^6 U/d	PDN 15 mg/d<br>Second-line therapy: Imatinib mesylate, mepolizumab.
Cardiovascular risk factors	Mild dyslipidemia.<br>Never smoker.	None.<br>Never smoker.	Non smoker.<br>Familial coronary disease: grand-fathers in their 60s.	None	NA
**Acute Serious Vascular Complication of HES**					
Age at time of event	52	62	38	18	NA
Delay since HE / HES diagnosis (yrs)	25/10	12/11	10/-	11/3	NA
Blood eosinophilia[a] (G/L)	8.18	2.08	3.6	0.99	NA
Presenting symptoms	Aphasia, right brachio-facial paresis	Acute chest pain at rest	Exertional chest pain, shortness of breath, nausea	Hypoesthesia left side of body and face, left-sided hearing loss	NA
Acute ischemic episode	Ischemic stroke	Acute coronary syndrome: NSTEMI	Acute coronary syndrome: NSTEMI	Ischemic stroke	Ischemic embolic stroke<br>Silent myocardial infarction
Underlying vascular anomalies	Spontaneous dissection of the left MCA	Multiple CA aneurysms, giant VS aneurysms with thrombotic material (likely source of CA embolism)	Multiple CA aneurysms with intra-luminal thrombosis	Spontaneous dissection of an aneurysm at the origin of the right PICA	Aneurysms of supra-aortic vessels (including left carotid bulb) and CA,<br>Thoracic aortic ectasia

Trans-thoracic echocardiography	Normal	Severe LV systolic dysfunction (ejection fraction 30-35%)	Apical hypokinesis	Normal	NA
Relevant assessments	Lumbar puncture: CSF nl.<br>Carotid Doppler US: moderate atheromatosis of the carotid bifurcation without stenosis; vertebral arteries normal.<br>Cerebral CT-angiography and MR: "flute-beak" stenosis of left MCA, cortico-subcortical left hemispheric infarction; vessel wall imaging sequences (MR): intramural hematoma of left MCA (T1 hyper-signal) consistent with arterial dissection.	ECG: normal<br>Troponin: 147 ng/L<br>Carotid Doppler US : mild atheromatosis at carotid bifurcation, vertebral arteries normal.<br>Coronary angiography: proximal aneurysmal dilatation of 3 main CA, extrinsic compression of left main trunk by VS.<br>Cardiac MR: subendocardial septo-apical LGE.<br>Aortic CT angiography: giant aneurysms of the left (3.8 cm) and non-coronary VS.<br>Per-operative TEE: thrombus in the VS giant aneurysm.	ECG: normal<br>Troponin: 1280 ng/L<br>Coronary angiography: aneurysms on left main CA, mid-LAD, ramus intermedius, first obtuse marginal branch; thrombus within aneurysmal segments<br>Cerebro-vascular CT angiography: aneurysm of basilar artery	Brain MR: restricted diffusion in the PICA territory (infarction)<br>Cerebral MR angiography: dissecting aneurysm at the origin of the right PICA	NA
Management of vascular event	ASA, atorvastatin	ED: ASA, clopidogrel, isosorbide dinitrate, unfractionated heparin.<br>Day 10: aortic root replacement by mechanical Bentall procedure with CABG.<br>Post-op: warfarin, bisoprolol	ED: ASA, nitroglycerine, hydromorphone<br><br>4-vessel CABG, ASA, clopidogrel, atorvastatin, metoprolol - anticoagulant	ASA, dipyridamol, statin	NA
Management of HES	Intensification of maintenance therapy.	Initiation of maintenance OCS therapy.	Initiation of maintenance therapy	No change in maintenance therapy	NA
Outcome	Regression of neurological deficit.<br>HE not controlled with MPDN 4 mg/d + HU or Peg-IFN-α.<br>Complete hematological response to mepolizumab 300 mg/4 wks.	Normalization of LV function and serum troponin.<br>Failure to maintain AEC in normal range with low-dose OCS. Plan to initiate mepolizumab.	Recanalization of thrombosed right CA, decrease in thrombus burden within aneurysmal segments. Preserved LV function.<br>OCS-resistant. Disease controlled with mepolizumab.	Hemisensory disturbances resolved within 1 week.<br>Unchanged PICA aneurysm 1 year later.<br>Maintenance treatment pursued with PDN 5 mg/d, IFNα $4x10^6$ U/d.	NA

AEC absolute eosinophil count, ASA acetylsalicylic acid, CA coronary artery, CABG coronary artery bypass graft, CSF cerebrospinal fluid, CT computed tomography, Cx circumflex, DUS Doppler ultrasound, ECG electrocardiogram, ED emergency department, EDN eosinophil derived

neurotoxin, eMBP1 eosinophil major basic protein, H&E: hematoxylin and eosin, HE hypereosinophilia, HES hypereosinophilic syndrome, HR hematological response, HU hydroxyurea, IIF indirect immuno-fluorescence, IFN interferon, IV intravenous, LAD left anterior descending, LGE late gadolinium enhancement, LV left ventricular, MCA middle cerebral artery, MMF mycophenolate mofetil, MPDN methylprednisolone, MR magnetic resonance, NA not available, NSTEMI non ST elevation myocardial infarction, OCS oral corticosteroid, PDN prednisolone, Peg pegylated, PICA postero-inferior cerebellar artery, TEE trans-esophageal echocardiography, US ultrasound, VS Valsalva sinus, wk week, yr year

[a] With the exception of patient from reference 9 for whom data is not available, all patients had persistently elevated eosinophil counts during the period between detection of HE (or diagnosis of HES) and occurrence of the acute ischemic event, either because they were not or sub-optimally treated.

Supplement S2

- (((Hypereosinophilic syndrome [MeSH Terms]) AND ((aneurysm [MeSH Terms]) OR (acute stroke [MeSH Terms]) OR (artery dissection [MeSH Terms]))
- Lymphoid variant Hypereosinophilic syndrome [MeSH Terms] AND (outcome [MeSH Terms] OR clinical manifestations [MeSH Terms]))
